# Meniscal extrusion promotes knee osteoarthritis structural progression: protective effect of strontium ranelate treatment in a phase III clinical trial

**DOI:** 10.1186/s13075-015-0579-4

**Published:** 2015-03-23

**Authors:** Camille Roubille, Johanne Martel-Pelletier, Jean-Pierre Raynauld, François Abram, Marc Dorais, Philippe Delorme, Jean-Pierre Pelletier

**Affiliations:** Osteoarthritis Research Unit, University of Montreal Hospital Research Centre (CRCHUM), 900 Saint-Denis, Pavillon R, Suite R11.412A, Montreal, Quebec H2X 0A9 Canada; Medical Imaging Research & Development, ArthroLab Inc, Montreal, Quebec Canada; StatSciences Inc, Notre-Dame-de-l’Île-Perrot, Quebec Canada

## Abstract

**Introduction:**

To evaluate the impact of meniscal extrusion (Ext) on knee osteoarthritis (OA) structural progression and on response to strontium ranelate (SrRan) treatment at 36 months in patients with (+) or without (-) Ext, in association (+) or not (-) with bone marrow lesions (BML) in the medial compartment using X-rays (JSW) and qMRI.

**Methods:**

Patients from the qMRI substudy of the SEKOIA trial (SrRan 1 g/day, n = 113; SrRan 2 g/day, n = 105; placebo, n = 112) were stratified based on whether meniscal extrusion and/or BML were present or not in the medial compartment.

**Results:**

In the placebo group, Ext+ patients (n = 26) had more JSW loss (p = 0.002) and cartilage volume loss in the global knee (p = 0.034) and plateau (p = 0.005), and medial compartment (p = 0.0005) than Ext- patients (n = 86). Ext-BML+ patients (n = 18) demonstrated more JSW loss (p = 0.003) and cartilage volume loss in the global (p = 0.020) and medial femur (p = 0.055) than Ext-BML- (n = 68). Compared to Ext+ BML- (n = 14), Ext+ BML+ patients (n = 12) had more cartilage volume loss in the global femur (p = 0.028), with no change in JSW. The JSW loss (p = 0.0004) and cartilage volume loss (global knee, p = 0.033, medial compartment, p = 0.0005) were greater when Ext and BML were simultaneously present in the medial compartment. SrRan 2 g/day treatment demonstrated a reduction in OA knee structural progression with qMRI, but not with JSW, in which less cartilage volume loss was found in the plateaus (p = 0.007) in Ext+ patients (n = 15), and in the medial plateau (p = 0.046) in patients in whom both Ext and BML were co-localized.

**Conclusion:**

The findings of this study are novel and could have an impact on future strategies regarding clinical trials. Indeed, data first argue for a combined, cumulative effect of meniscal extrusion and bone marrow lesions on cartilage loss and, secondly, they showed that SrRan may have protective effects in OA patients with meniscal extrusion as well as when both meniscal extrusion and BML are co-localized.

## Introduction

Knee osteoarthritis (OA) structural changes are complex and comprise cartilage loss as well as meniscal lesions, including extrusion [1,2]. These are believed to not only be responsible for disease symptoms but also to promote cartilage degeneration and loss [3]. To date, joint space width (JSW) loss over time is still considered the gold standard to evaluate the effects of disease-modifying OA drugs (DMOAD) [2]. However, the integrity of surrounding tissues, particularly the meniscus, may affect the reliability of such measurement [4]. Moreover, quantitative magnetic resonance imaging (qMRI) has been shown to be instrumental in identifying factors predictive of treatment responsiveness, such as bone marrow lesions (BML) [2,5].

We and others have shown that the presence of severe meniscal extrusion is predictive of knee OA structural progression as assessed by qMRI, and a strong predictor of cartilage volume loss over time [3,6-11]. Another important risk factor for OA structural progression is the presence of BML [11,12]. Although a recent study using semiquantitative MRI scoring for cartilage assessment showed that the risk of cartilage loss is increased with co-localized pathologies (meniscal extrusion (Ext) and BML) and further increased when more than one such pathology is present [13], these findings need further confirmation by radiography and qMRI.

In the context of personalized management of OA, another clinically relevant concern is the impact of meniscal extrusion, alone or in conjunction with BML, on the response to putative DMOAD treatment. This question was raised by the findings of recent studies using qMRI, in which patients with severe meniscal extrusion demonstrated more severe cartilage volume loss but were also more responsive to such treatment [14], and in a subset of patients from the phase III knee OA trial (SEKOIA) showing that Strontium ranelate (SrRan) (2 g/day) at 36-month follow up significantly decreased cartilage volume loss in the medial plateau in patients with BML in the medial compartment [15]. However, the impact of the simultaneous presence of meniscal extrusion and BML on response to treatment that may modify disease structural progression is at present unknown and is an issue of great clinical interest.

To address these issues, we analyzed patients from a sample of the phase III knee OA SEKOIA trial [16] used in a recent qMRI study [15] and evaluated the impact of extrusion at baseline, co-localized or not with BML in the medial compartment, on knee OA structural progression in the placebo group, and on response to SrRan treatment at 36-month follow up, comparing JSW loss (radiography) and cartilage volume loss (qMRI) in the global knee and subregions, with emphasis on the medial compartment.

## Methods

### Study sample and treatment

The patient sample was as described in our recent report [15]. Briefly, 438 patients from the three-year SEKOIA cohort were randomized to undergo MRI examinations. The modified intention-to-treat (mITT) sample (n = 330) included all randomized patients who received at least one dose of SrRan and had at least two MRI examinations as described (placebo, n = 112; SrRan 1 g/day, n = 113; SrRan 2 g/day, n = 105) [15]. The protocol and other documents of the original SEKOIA study [16] related to informed consent and investigator information were reviewed by independent ethics committees in the countries concerned and by the investigators and coordinators in accordance with local regulatory requirements. Written informed consent was obtained from all participants. The study was performed in accordance with the ethical principles laid out in the Declaration of Helsinki and is registered (ISRCTN41323372).

### Study design

The patients of the three groups were stratified into two main groups based on the presence (+) or absence (-) of Ext in the medial compartment at baseline, and further stratified based on the presence (+) or absence (-) of BML in the medial compartment, co-localized or not, with Ext. This stratification was based on regrouping patients with similar knee OA risk factors at baseline that are predictive of structural progression. Ext was assessed using the sagittal three-dimensional intermediate-weighted sequence with fat suppression (VISTA-SPAIR), which allows for a precise evaluation of the extent of the extrusion, and scored as absence or presence of partial or complete extrusion detected in any of the three segments of the meniscus as described [3,9]. BMLs were assessed with the sagittal proton density-weighted fast spin-echo sequence with fat suppression (PD-FSE) and evaluated as absence or presence as described [17].

### Imaging of the knee

MRI scans were performed at baseline and at 36 months using a 1.5-Tesla scanner (Siemens, Erlangen, Germany or General Electric, Milwaukee, WI, USA) with a dedicated knee coil, as previously described [15]. The cartilage volume and change were measured using the proprietary software, Cartiscope™ (ArthroLab, Montreal, QC, Canada) [18]. The minimal JSW (mm) at the narrowest point in the medial tibiofemoral compartment was determined by radiography and measured as described [15].

### Symptoms

Disease symptoms were assessed at baseline as described, using the Western Ontario and McMaster Universities Osteoarthritis Index (WOMAC) questionnaire and the visual analog scale (VAS) for global knee pain [15].

### Outcomes

The first endpoint of the study was the change in JSW and in cartilage volume in the medial compartment at 36 months in the placebo group according to the absence or presence of Ext and/or BML in the medial compartment at baseline. In this study, emphasis was on the assessment of cartilage volume in the medial compartment as this area is related to JSW measurement. The second endpoint was to assess and compare change in the JSW and cartilage volume in the medial compartment at 36 months in the SrRan treatment groups versus placebo, according to the presence of Ext and/or BML in the medial compartment at baseline.

### Statistical analyses

The analyses of the mITT patients (n = 330) were carried out by imputing the missing data to the average change recorded (mean value imputed) among patients within their corresponding group. Within the placebo group, we compared the baseline demographic, clinical, and imaging characteristics discriminating Ext+ and Ext-, and subgroups BML+ and BML-, using the Kruskall-Wallis test (non-normal distribution) for continuous variables or the chi-square test for categorical variables. The relationship between the number of co-localized factors (Ext and/or BML) and JSW loss and cartilage volume loss in the medial compartment at 36 months was assessed by analysis of covariance (ANCOVA) using the number of co-localized factors in the medial compartment at baseline as dependent variable (0 = no Ext or BML, 1 = either Ext or BML, 2 = Ext and BML). Further comparisons between the three treatment groups were performed using the Kruskall-Wallis test or the chi-square test. When the *P*-values were <0.10, each treatment group was further analyzed as two-by-two comparison using the Mann-Whitney test or the chi-square test with Bonferroni adjustment. The change in JSW and cartilage volume was also analyzed using multivariate regression models when appropriate and, provided that samples were large enough, adjusting for potential confounding factors at baseline.

## Results

### Study sample and baseline characteristics

**Figure 1:**
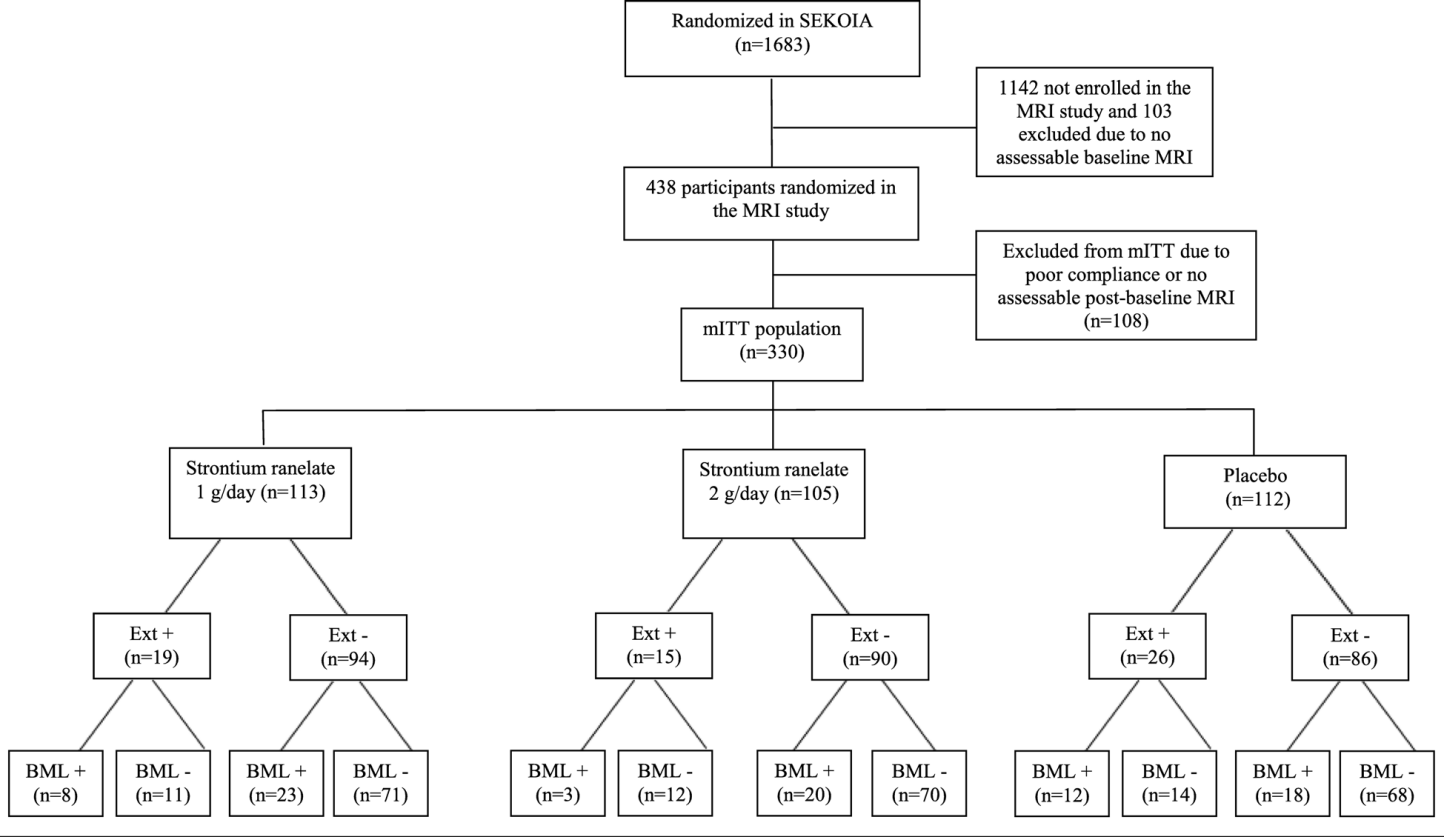
**Study design.** n, number of participants; MRI; magnetic resonance imaging; mITT, modified intention-to-treat; Ext, meniscal extrusion; BML, bone marrow lesions.

Baseline characteristics of the entire mITT sample have been previously described [15]. In this post hoc analysis, the Ext+ group comprised 60 patients (placebo, n = 26; SrRan 1 g/day, n = 19; SrRan 2 g/day, n = 15), and the Ext- group, 270 patients (placebo, n = 86; SrRan 1 g/day, n = 94; SrRan 2 g/day, n = 90) (Figure 1).

#### Baseline characteristics within the placebo group

In the placebo group (n = 112), demographic and clinical characteristics were balanced between Ext+ (n = 26) and Ext- patients (n = 86) (Table 1). However, Ext+ patients had more severe structural disease at baseline as assessed by radiography, showing significantly narrower JSW and more frequent grade-3 Kellgren-Lawrence (KL) scores. No differences between these groups were found in cartilage volume when assessed by qMRI (Table 1). However, there were significantly more Ext+ patients with BML+ at baseline (Table 1).

**Table 1:** **Baseline demographic, clinical, and imaging characteristics of the placebo group according to the absence or presence of meniscal extrusion**

	**Ext- (n = 86)**	**Ext+ (n = 26)**	***P*** **-value**
**Demographic and clinical**			
Age, years	62 ± 8	65 ± 8	0.078
Female, n (%)	57 (66%)	19 (73%)	0.515^†^
Body mass index, kg/m^2^	30 ± 4	29 ± 6	0.275
WOMAC			
Pain (0 to 100)	37.7 ± 21.9	40.9 ± 20.0	0.340
Function (0 to 100)	39.5 ± 22.4	38.3 ± 22.7	0.762
Stiffness (0 to 100)	41.9 ± 24.5	46.4 ± 19.4	0.403
Total (0 to 300)	120.6 ± 65.0	127.1 ± 56.6	0.654
VAS pain (0 to 100 mm)	48.5 ± 25.2	54.3 ± 18.8	0.199
**Imaging**			
Kellgren-Lawrence score, n (%)			**0.002** ^**†**^
Grade 1	-	-	
Grade 2	64 (74%)	11 (42%)	
Grade 3	22 (26%)	15 (58%)	
Joint space width, mm	3.61 ± 0.77	3.15 ± 0.51	**0.004**
Presence of BML in the medial compartment, n (%)	18 (21%)	12 (46%)	**0.011** ^**†**^
MRI, mm^3^			
**Global knee**	11256 ± 2968	10479 ± 2243	0.395
Femur	7773 ± 2139	7398 ± 1465	0.697
Plateau	3483 ± 897	3081 ± 830	0.109
**Medial compartment**	5324 ± 1435	5089 ± 1020	0.682
Medial femur	3763 ± 1023	3619 ± 689	0.817
Medial plateau	1562 ± 452	1470 ± 360	0.608

Results are shown as mean ± SD unless otherwise indicated. ^†^ *P*-values obtained using the chi-square test. All other *P*-values were obtained using the Kruskal-Wallis test. Statistically significant P-values are in bold. Ext, meniscal extrusion; n, number of participants; WOMAC, Western Ontario and McMaster Universities Osteoarthritis Index (each subscale, 100 = worst score; total scale, 300 = worst score). ; VAS, visual analog scale (0 mm= no pain, 100 mm= most severe pain); MRI, magnetic resonance imaging; BML, bone marrow lesions.

When stratifying Ext- and Ext+ groups based on the co-localization of BML (Table 2), the Ext-BML+ patients had higher KL grades than those without BML. No differences were found in JSW or cartilage volume between groups. Yet, WOMAC total score, function and pain were higher in the Ext+ BML- group than in the Ext+ BML+ group (Table 2). Interestingly, although not statistically significant, cartilage volume in all knee regions studied was lower in the Ext+ BML+, and compared to the Ext+ BML- group, numerical trends were found in the medial compartment (*P* = 0.095) and medial femur (*P* = 0.068). Likewise, Ext+ BML+ patients had the narrowest JSW among all subgroups, but the difference compared to Ext+ BML- did not reach statistical significance.

**Table 2:** **Baseline demographic, clinical, and imaging characteristics of the placebo group according to absence or presence of meniscal extrusion and bone marrow lesions (modified intention-to-treat sample, n = 330)**

	**Ext- (n = 86)**			**Ext+ (n = 26)**		
	**BML- (n = 68)**	**BML+ (n = 18)**	***P*** **-value**	**BML- (n = 14)**	**BML+ (n = 12)**	***P*** **-value**
**Demographic and clinical**						
Age, years	61 ± 7	64 ± 10	0.493	65 ± 7	65 ± 10	>0.999
Female, n (%)	47 (69%)	10 (56%)	0.279^†^	9 (64%)	10 (83%)	0.275^†^
Body mass index, kg/m^2^	30 ± 4	30 ± 3	0.824	31 ± 7	27 ± 3	0.116
WOMAC						
Pain (0 to 100)	37.4 ± 22.7	39.2 ± 19.0	0.587	48.5 ± 19.2	32.8 ± 18.2	**0.053**
Function (0 to 100)	40.7 ± 22.5	35.5 ± 22.3	0.449	46.6 ± 22.6	28.7 ± 19.5	**0.021**
Stiffness (0 to 100)	41.4 ± 26.3	43.7 ± 16.3	0.834	50.8 ± 17.5	41.4 ± 21.1	0.208
Total (0 to 300)	120.9 ± 67.5	119.6 ± 55.8	0.871	149.4 ± 51.5	102.9 ± 53.5	**0.024**
VAS pain (0 to 100 mm)	50.4 ± 25.6	41.3 ± 23.1	0.126	56.9 ± 17.2	51.3 ± 20.8	0.425
**Imaging**						
Kellgren-Lawrence, n (%)			**0.039** ^**†**^			0.391^†^
Grade 1	-	-		-	-	
Grade 2	54 (79%)	10 (56%)		7 (50%)	4 (33%)	
Grade 3	14 (21%)	8 (44%)		7 (50%)	8 (67%)	
Joint space width, mm	3.68 ± 0.74	3.37 ± 0.85	0.125	3.28 ± 0.55	2.99 ± 0.42	0.207
MRI, mm^3^						
**Global knee**	11247 ± 3151	11292 ± 2218	0.622	10930 ± 1749	9952 ± 2694	0.190
Femur	7725 ± 2264	7956 ± 1621	0.321	7748 ± 1225	6989 ± 1664	0.129
Plateau	3522 ± 943	3336 ± 700	0.803	3182 ± 591	2962 ± 1060	0.456
**Medial compartment**	5334 ± 1525	5288 ± 1065	0.746	5355 ± 841	4479 ± 1155	0.095
Medial femur	3766 ± 1089	3752 ± 750	0.549	3818 ± 550	3388 ± 783	0.068
Medial plateau	1568 ± 471	1537 ± 384	>0.999	1538 ± 336	1390 ± 384	0.247

Results are shown as mean ± SD unless otherwise indicated. ^†^ *P*-values obtained using the chi-square test. All other *P*-values were obtained using the Kruskal-Wallis test. Statistically significant P-values are in bold. Ext, meniscal extrusion; n, number of participants; BML, bone marrow lesions; WOMAC, Western Ontario and McMaster Universities Osteoarthritis Index (each subscale, 100 = worst score; total scale, 300 = worst score); VAS, visual analog scale (0 mm = no pain, 100 mm = most severe pain); MRI, magnetic resonance imaging.

#### Baseline characteristics within the treatment groups

The SrRan 1 g/day, 2 g/day and placebo groups were fairly balanced at baseline except for age in the Ext- patients and, in the Ext+ patients, gender and lower global knee cartilage volume in the placebo group (Table 3).

**Table 3:** **Baseline demographic, clinical, and imaging characteristics in the treatment groups according to the absence or presence of meniscal extrusion (Ext)**

	**Strontium ranelate 1 g/day**	**Strontium ranelate 2 g/day**	**Placebo**	***P*** **-value**	**Placebo versus Strontium ranelate 1 g/day**	**Placebo versus Strontium ranelate 2 g/day**
					***P*** **-value**	***P*** **-value**
**Ext-**	**n = 94**	**n = 90**	**n = 86**			
**Demographic and clinical**						
Age, years	60 ± 7	63 ± 7	62 ± 8	0.045*	0.961**	0.548**
Female, n (%)	63 (67%)	61 (68%)	57 (66%)	0.978^†^		
Body mass index, kg/m^2^	30.0 ± 5.0	29.8 ± 9.9	32.4 ± 8.1	0.187*		
WOMAC						
Pain (0 to100)	40 ± 20	44 ± 21	38 ± 22	0.107*		
Function (0 to 100)	42 ± 21	42 ± 24	40 ± 22	0.732*		
Stiffness (0 to 100)	46 ± 23	48 ± 26	42 ± 24	0.294*		
Total (0 to 300)	130 ± 59	132 ± 64	121 ± 65	0.446*		
VAS pain (0 to 100 mm)	50 ± 22	54 ± 24	48 ± 25	0.204*		
**Imaging**						
Kellgren-Lawrence, n (%)				0.943^†^		
Grade 1	-	-	-			
Grade 2	71 (76%)	66 (73%)	64 (74%)			
Grade 3	23 (24%)	24 (27%)	22 (26%)			
Joint space width, mm	3.61 ± 0.82	3.67 ± 0.81	3.61 ± 0.77	0.707*		
Presence of BML, n (%)	23 (24%)	20 (22%)	18 (21%)	0.847^†^		
MRI, mm^3^						
**Global knee**	11256 ± 2920	11520 ± 3344	11256 ± 2968	0.911*		
Femur	7759 ± 2121	8000 ± 2286	7773 ± 2139	0.750*		
Plateau	3497 ± 886	3520 ± 1158	3483 ± 897	0.855*		
**Medial compartment**	5391 ± 1374	5547 ± 1577	5325 ± 1435	0.655*		
Medial femur	3797 ± 1020	3937 ± 1098	3763 ± 1023	0.540*		
Medial plateau	1594 ± 405	1610 ± 541	1562 ± 452	0.726*		
**Ext+**	**n = 19**	**n = 15**	**n = 26**			
**Demographic and clinical**						
Age, years	61 ± 6	61 ± 8	65 ± 8	0.228*		
Female, n (%)	7 (37%)	9 (60%)	19 (73%)	**0.051** ^†^	0.045**	>0.999**
Body mass index, kg/m^2^	30.7 ± 4.7	32.2 ± 6.4	29.3 ± 6.0	0.164*		
WOMAC						
Pain (0 to100)	50 ± 21	50 ± 16	41 ± 20	0.320*		
Function (0 to 100)	47 ± 21	45 ± 19	38 ± 23	0.593*		
Stiffness (0 to 100)	52 ± 24	57 ± 17	46 ± 19	0.252*		
Total (0 to 300)	147 ± 58	153 ± 46	127 ± 57	0.394*		
VAS pain (0 to 100 mm)	56 ± 19	58 ± 19	54 ± 19	0.837*		
**Imaging**						
Kellgren-Lawrence, n (%)				0.158†		
Grade 1	-	-	-			
Grade 2	6 (32%)	2 (13%)	11 (42%)			
Grade 3	13 (68%)	13 (87%)	15 (58%)			
Joint space width (mm)	2.91 ± 0.75	2.80 ± 0.35	3.15 ± 0.51	0.110*		
Presence of BML, n (%)	8 (42%)	3 (20%)	12 (46%)	0.232^†^		
MRI, mm^3^						
**Global knee**	12345 ± 3367	12838 ± 2972	10479 ± 2243	0.032*	0.193**	0.050**
Femur	8497 ± 2192	8962 ± 2100	7398 ± 1465	0.044*	0.274**	0.062**
Plateau	3848 ± 1281	3875 ± 1051	3081 ± 830	0.029*	0.100**	0.071**
**Medial compartment**	5707 ± 1520	5845 ± 1305	5089 ± 1020	0.208*		
Medial femur	4013 ± 958	4136 ± 945	3619 ± 689	0.176*		
Medial plateau	1695 ± 624	1708 ± 397	1470 ± 360	0.295*		

Results are shown as mean ± SD unless otherwise indicated. *P-values assessed using the Kruskal-Wallis test; **P-values assessed using the **Mann-Whitney test with Bonferroni adjustment for multiple comparisons; *P*-values assessed using the †chi-square test for categorical variables. Statistically significant P-values are in bold. Ext, meniscal extrusion; n, number of participants; WOMAC, Western Ontario and McMaster Universities Osteoarthritis Index (each subscale, 100 = worst score; total scale, 300 = worst score); VAS, visual analog scale (0 mm = no pain, 100 mm = most severe pain); BML, bone marrow lesions MRI, magnetic resonance imaging.

Co-localization or not with BML revealed no differences except for body mass index (BMI) and JSW in the Ext+ BML+ patients, in which a higher BMI was found in the SrRan 1 g/day group versus placebo (*P* = 0.003), and less JSW in the SrRan 1 g/day group versus placebo (*P* = 0.015) (data not shown).

### Change in JSW and cartilage volume at 36 months in the placebo group

Compared to Ext-, the Ext+ patients demonstrated significantly more JSW loss as analyzed using univariate analysis (Table 4). This result was further confirmed in multivariate regression (*P* = 0.021) (data not shown) adjusted for the features that were different at baseline (see Table 1): KL grade, presence of BML, and JSW.

**Table 4:** **Joint space width loss and cartilage volume loss at 36 months in the placebo group according to the co-localization of meniscal extrusion and bone marrow lesions in the medial compartment at baseline**

	**Ext- (n = 86)**			**Ext+ (n = 26)**		***P*** **-value**
**Joint space width loss**						
mm	-0.35 ± 0.61			-0.76 ± 0.67		**0.002**
%	-10.36 ± 20.10			-23.24 ± 19.23		**0.0008**
**MRI (%)**						
**Global knee**	-6.70 ± 2.78			-8.02 ± 3.28		**0.034**
Femur	-6.15 ± 3.29			-7.28 ± 2.91		0.171
Plateau	-7.80 ± 5.05			-10.01 ± 5.79		**0.005**
**Medial compartment**	-7.60 ± 4.63			-10.40 ± 4.23		**0.0005**
Medial femur	-7.90 ± 4.76			-10.89 ± 3.19		**0.0002**
Medial plateau	-6.77 ± 7.52			-9.07 ± 8.74		**0.012**
	**BML- (n = 68)**	**BML+ (n = 18)**	***P*** **-value**	**BML- (n = 14)**	**BML+ (n = 12)**	***P*** **-value**
**Joint space width loss**						
mm	-0.23 ± 0.55	-0.77 ± 0.68	**0.003**	-0.88 ± 0.82	-0.61 ± 0.43	0.455
%	-5.79 ± 15.35	-27.64 ± 26.29	**0.002**	-25.44 ± 22.79	-20.68 ± 14.62	0.589
**MRI (%)**						
**Global knee**	-6.75 ± 2.81	-6.50 ± 2.71	0.227	-6.89 ± 3.70	-9.33 ± 2.19	0.066
Femur	-6.39 ± 3.36	-5.26 ± 2.92	**0.020**	-6.16 ± 2.97	-8.57 ± 2.32	**0.028**
Plateau	-7.38 ± 4.15	-9.40 ± 7.49	0.162	-8.67 ± 7.12	-11.57 ± 3.37	0.093
**Medial compartment**	-7.08 ± 4.33	-9.57 ± 5.29	0.090	-9.69 ± 5.50	-11.22 ± 1.90	0.103
Medial femur	-7.54 ± 4.90	-9.26 ± 4.03	**0.055**	-10.72 ± 4.08	-11.08 ± 1.86	0.339
Medial plateau	-5.79 ± 5.68	-10.46 ± 11.70	0.185	-7.04 ± 10.97	-11.45 ± 4.47	0.155

Results for cartilage volume loss are percentage (%) of change expressed as mean ± SD. *P*-values were assessed using the Kruskal-Wallis test. Statistically significant P-values are in bold. Ext, meniscal extrusion; n, number of participants; MRI, magnetic resonance imaging; BML, bone marrow lesions.

Moreover, the Ext+ patients had significantly more cartilage volume loss in the global knee and plateau, and in the medial compartment including both femur and plateau (Table 4). In multivariate analysis adjusted as described above, a numerical trend (*P* = 0.12) toward more cartilage volume loss in the medial compartment in Ext+ patients was found with statistical significance at the medial femur (*P* = 0.053) (data not shown). Data further showed that the Ext+ patients also had more cartilage volume loss in the lateral plateau than the Ext- patients (-10.90% ± 5.08 versus -8.57% ± 4.95, *P* = 0.011).

Evaluation of the impact of co-localization of Ext and BML on change in JSW and cartilage volume revealed that in the Ext- group, BML+ patients had significantly more loss of JSW and of cartilage volume in the global and medial femur with a numerical trend in the medial compartment compared with BML- patients (Table 4). Of note, although not reaching statistical significance, Ext-BML+ patients showed higher cartilage volume loss (about 55%) in the medial plateau than the Ext-BML- patients.

In the Ext+ group, while BML+ and BML- patients were not different with regard to JSW loss, BML+ patients demonstrated more cartilage volume loss in the global femur with trends in the global knee and in the medial compartment (Table 4). Interestingly, assessment of JSW loss and cartilage volume loss in the medial compartment according to the number of co-localized factors (Ext ± BML) at baseline (Table 5) revealed that loss of both JSW and cartilage volume in the global knee and all subregions studied except the global femur, significantly increased with an additional co-localized factor (that is, from no factors to one factor, or from one factor to two factors). Hence, JSW and cartilage volume loss were greater when Ext and BML were co-localized.

**Table 5:** Impact of co-localized factors* present in the medial compartment at baseline on loss of joint space width and of cartilage volume at 36 months in the placebo group

	**Loss of joint space width and cartilage volume linked to addition of one co-localized factor**	***P*** **-value**
**Joint space width loss**		
mm	0.31	**0.0004**
%	-11.58	**<0.0001**
**MRI (%)**		
**Global knee**	-0.86	**0.033**
Femur	-0.53	0.239
Plateau	-1.97	**0.007**
**Medial compartment**	-2.22	**0.0005**
Medial femur	-1.95	**0.002**
Medial plateau	-2.93	**0.006**

*P*-values were assessed with analysis of covariance (ANCOVA) using the number of co-localized factors in the medial compartment at baseline as the dependent variable. Statistically significant P-values are in bold. *Co-localized factors: 0 = neither bone marrow lesions nor meniscal extrusion, 1 = either meniscal extrusion or bone marrow lesions, 2 = meniscal extrusion and bone marrow lesions. MRI, magnetic resonance imaging.

### Effect of SrRan treatment at 36 months

Because the main aim of this work was to understand the various effects of Ext and BML on JSW and cartilage volume changes, the evaluation of the response to SrRan treatment was performed on the Ext+ subjects only (Table 6). Data revealed no statistically significant difference between treatment groups for JSW loss. However, SrRan at 2 g/day demonstrated a significant reduction in cartilage volume loss in the global plateau in both univariate (*P* = 0.007, Table 6) and multivariate analyses (*P* = 0.014, data not shown), the latter adjusted for gender and cartilage volume which were significantly different at baseline and considered as potential confounding factors. Moreover, although SrRan 2 g/day markedly decreased (by about 66%) the cartilage volume loss in the medial plateau, the difference did not quite reach statistical significance but yielded a numerical trend (*P* = 0.081, Table 6), probably due to the relatively small number of patients in each group. Interestingly, the Ext+ patients demonstrated that both SrRan 1 g/day and 2 g/day significantly reduced the cartilage volume loss in the lateral plateau (-7.29% ± 3.86, *P* = 0.019 and -6.46% ± 3.93, *P* = 0.014, respectively) compared to placebo (-10.90% ± 5.08).

**Table 6:** **Loss of joint space width and of cartilage volume at 36 months in treatment groups in patients with meniscal extrusion**

	**SrRan 1 g/day**	**SrRan 2 g/day**	**Placebo**	***P*** **-value**	**Placebo versus ** **SrRan 1 g/day**	**Placebo versus ** **SrRan 2 g/day**
	**n = 19**	**n = 15**	**n = 26**		***P*** **-value**	***P*** **-value**
**Joint space width loss**						
mm	-0.46 ± 0.45	-0.41 ± 0.42	-0.76 ± 0.67	0.085*		
%	-18.54 ± 19.69	-14.67 ± 15.69	-23.24 ± 19.23	0.228*		
**MRI (%)**						
**Global knee**	-7.66 ± 2.10	-6.67 ± 2.56	-8.02 ± 3.28	0.351*		
Femur	-7.70 ± 2.66	-6.98 ± 4.26	-7.28 ± 2.91	0.809*		
Plateau	-7.61 ± 3.79	-5.74 ± 3.54	-10.01 ± 5.79	0.003*	0.070**	0.007**
**Medial compartment**	-10.29 ± 3.68	-9.14 ± 3.27	-10.40 ± 4.23	0.119*		
Medial femur	-11.17 ± 4.25	-10.72 ± 5.36	-10.89 ± 3.19	0.290*		
Medial plateau	-8.13 ± 5.98	-4.90 ± 5.19	-9.07 ± 8.74	0.048*	0.708**	0.081**

Results for cartilage volume loss are percentage (%) of change expressed as mean ± SD. *P-values assessed using the Kruskal-Wallis test; *P*-values assessed using the **Mann-Whitney test with Bonferroni adjustment. Statistically significant P-values are in bold. SrRan, strontium ranelate; n, number of participants; MRI, magnetic resonance imaging.

Further analysis in Ext+/BML- patients showed no significant difference between SrRan and placebo with regard to the loss of JSW and of cartilage volume. Although subgrouping results in a lower number of patients in each group, interesting data were found only for qMRI when Ext and BML were co-localized. Hence, Ext+ BML+ patients in the SrRan 1 g/day group demonstrated a significant reduction in cartilage volume loss in the global plateau (*P* = 0.017) and in the SrRan 2 g/day group in the medial plateau (*P* = 0.046), compared with placebo (data not shown).

## Discussion

The present study aimed at expanding our previous findings [15] and to evaluate the impact of meniscal extrusion, co-localized or not with BML in the medial compartment, on the natural structural progression of knee OA using the placebo group, and on the response to SrRan by comparing the loss of JSW and of cartilage volume.

In the placebo group, the proportion of patients with meniscal extrusion was lower than in some studies [9,18], reinforcing our assumption in the previous report on this trial [15] that this cohort had a more moderate degree of disease than those in other clinical trials [14]. However, the global and medial cartilage volume changes in the patients with meniscal extrusion are in line with the findings of previous studies [2,9]. Showing that meniscal extrusion was associated with more rapid progression of knee OA as evaluated by both radiography and qMRI met our first outcome, further confirming previous results [3,8-11,13,14]. These data are also consistent with previous reports [15,19,20] of a positive correlation between loss of JSW and of cartilage volume in the medial compartment.

Meniscal extrusion has been formerly found to contribute to joint space narrowing [21,22] and it has been suggested that the meniscal position, rather than a real thinning of articular cartilage, is involved [4,21]. In this study, data demonstrated that the cartilage volume loss was factual, reinforcing the causal relationship between meniscal extrusion and cartilage loss [7,23-25]. The finding that patients with meniscal extrusion had more cartilage volume loss in the lateral plateau, in addition to being an important add-on to radiographic data showing that meniscal extrusion in the medial compartment could not only induce more cartilage volume loss in the ipsilateral compartment but also act in the contralateral one, suggests a triggering of pathways leading to cartilage damage in the whole joint. One may argue that meniscal extrusion likely generates subchondral bone remodeling and BML, which subsequently lead to cartilage loss [25]. However, given that the association between meniscal extrusion and JSW loss remained significant after adjusting for the presence of BML at baseline, meniscal extrusion in itself seems to independently impact the structural progression.

Rather than a greater impact of meniscal extrusion on BML or the reverse (BML on the extrusion), the literature indicates that the co-localization of these lesions is of greater importance for knee OA structural progression [26,27]. Here, only data with qMRI agree with this statement. While the presence of BML in patients without meniscal extrusion was a risk factor for progression as assessed by either JSW or cartilage volume, in patients with meniscal extrusion the presence of BML revealed no difference in JSW loss, but with qMRI, in addition to significant results in the global femur, statistical trends were found for change in cartilage volume in the global knee and plateau, and medial compartment. These data thus suggest that the JSW loss measurement is less sensitive than qMRI in evaluating the added impact of BML on OA structural progression. However, with regard to the radiographic data, one must take into account that this is a technique of low sensitivity and that the small number of patients in the subgroups may have reduced the strength of the analysis and explain the non-significant difference. Nevertheless, a cumulative negative impact of the presence of meniscal extrusion and BML at baseline on the loss of JSW and of cartilage volume in the medial compartment was demonstrated by covariance analyses showing that JSW loss was 0.31 mm and cartilage volume loss 2.22% per additional co-localized factor. This is the first demonstration of such an association using qMRI, but confirmed data from semiquantitative MRI showing that the risk of cartilage damage further increases when more than one associated co-localized pathology (meniscal extrusion, meniscal damage, BML) was present [13]. The present findings thus suggest that the presence of both meniscal extrusion and BML represent an additive risk for disease structural progression.

The higher level of symptoms (WOMAC pain, function) in the patients with meniscal extrusion but without BML compared to those with BML is intriguing. The extent to which BML contributes to knee OA symptoms in comparison to meniscal extrusion is still largely unknown. Although more extensive work needs to be done in order to answer this most relevant question, the data from a recent study [28] indicating that meniscal extrusion is the knee OA structural change most commonly associated with severe knee OA symptoms as well as the presence of neuropathic pain, may provide some explanation to the present finding.

With respect to the role of meniscal extrusion on the response to treatment, SrRan at 2 g/day significantly reduced the cartilage volume loss in the plateaus and in the medial plateau in the patients with both meniscal extrusion and BML. In addition to previous data showing that SrRan at 2 g/day can reduce cartilage volume loss in the medial plateau in patients with BML [15], data from this study suggest that SrRan may have structural protective effects in patients with both BML and meniscal extrusion, a condition which represents an even higher risk for more rapid structural disease progression [11,12,17,29]. The finding that in patients with meniscal extrusion, SrRan also reduced cartilage volume loss in the lateral plateau, where few or no BML were found [15], is interesting and reinforces findings that SrRan has a direct protective effect on cartilage loss [30,31] in addition to its impact on BML. This is supported by a previous report in a dog experimental OA model (anterior cruciate ligament model) showing that the protective effect of SrRan was associated with a reduction in the levels of pro-catabolic factors such as proteases and cytokines (IL-1β) in the diseased tissues [31]. Based on the present results, it can also be hypothesized that SrRan may protect against cartilage loss by both reducing BML and targeting the cartilage damage triggered by meniscal extrusion. In support of this hypothesis are the findings of a reduction in cartilage volume loss in patients with co-localized meniscal extrusion and BML. Together, the previous [15] and present data support a structural protective property of SrRan in patients with BML and meniscal extrusion in the medial compartment. These are clinically relevant, as cartilage volume loss in the medial compartment has been shown to be the most predictive of total knee replacement [2]. These results also support the added benefit of using qMRI as an alternative to radiography for the evaluation of putative DMOAD agents, especially in patients with less advanced disease [32] and provide important new insight into the role played by the concomitant risk factors (meniscal extrusion and BML) in the response of patients to OA treatments [2,14].

This study has limitations, the main one being the relatively small number of patients with meniscal extrusion, especially after stratification based on the co-localization of BML. In addition, the possible role of some other confounding factors, such as knee mal-alignment and prior meniscal resections, could not be evaluated as the information was not available. A complementary and interesting research question that could be addressed in further work is the assessment of bone area, which is also a potential confounding factor. Further MRI studies with a larger number of patients will be needed before any firm conclusions can be made, particularly with regard to the effect of SrRan on disease structural progression.

With regard to safety concerns, as reported in the original SEKOIA study [16,33], the number of treatment-emergent and serious treatment-emergent adverse events for SrRan 1 g/day, SrRan 2 g/day and placebo groups was similar between groups (85.8%, 87.9% and 86.5%, and 17.0%, 16.5% and 17.4%, respectively). No cases of drug reaction with eosinophilia and systemic symptoms, Stevens-Johnson syndrome, or toxic epidermal necrolysis were reported. Notably, because SrRan was found to be associated with an increased risk of myocardial infarction (MI) in osteoporosis trials, the European Medicine Agency defined new cardiovascular contraindications to SrRan. In the SEKOIA trial, five cases of serious MI were reported in the SrRan 2 g/day group, one case in the SrRan 1 g/day group, and in the placebo group, in patients with major cardiovascular risk factors or comorbidities. However, when applying a posteriori the newly defined contraindications of the European Medicine Agency to the SEKOIA population, the number of MIs was comparable between the three treatment groups: one event in the SrRan 1 g/day group, two in the SrRan 2 g/day group and one in the placebo group [33]. In any case, based on the current safety evidence, and consistent with the precautionary principle, SrRan cannot currently be recommended as a first-line DMOAD in knee OA patients.

## Conclusion

This study is complementary to the previous publication [15] and underlines the relevance of stratifying patients according to the absence or presence of meniscal extrusion. The results of the study are of great importance from two different perspectives: first, they argue for a combined, cumulative effect of meniscal extrusion and subchondral bone remodeling on cartilage damage and, second, they show that SrRan may have protective effects in knee OA patients with meniscal extrusion, even when co-localized with BML. Our findings are novel and could have an impact on strategies for the conduct of future trials, as well as supporting further research on potential structural effects of other bone anti-remodelling medications. This study also highlights that the response to treatment of knee OA patients is a complex issue, as it can be greatly influenced by the presence or absence of risk factors associated with disease structural progression.

## Abbreviations

BMI: body mass index
BML: bone marrow lesions
DMOAD: disease-modifying osteoarthritis drug
Ext: meniscal extrusion
JSW: joint space width
IL: interleukin
KL: Kellgren-Lawrence
MI: myocardial infarction
mITT: modified intention-to-treat
OA: osteoarthritis
qMRI: quantitative magnetic resonance imaging
SrRan: Strontium ranelate
VAS: visual analog scale
WOMAC: Western Ontario and McMaster Universities Osteoarthritis Index
